# Integrative medicine and health in undergraduate and postgraduate medical education

**DOI:** 10.3205/zma001442

**Published:** 2021-02-15

**Authors:** Eckhart Georg Hahn

**Affiliations:** 1 Friedrich-Alexander-University Erlangen-Nürnberg, University Hospital Erlangen, Department of Medicine 1, Erlangen, Germany

**Keywords:** undergraduate medical education, postgraduate medical education, integrative medicine and health, evidence-based medicine, evidence-based healthcare, medical roles, medical competences, National Competence-based Catalogue of Learning Objectives for Germany (NKLM 2.0), Medical Licensure Act in Germany (ÄApprO)

## Abstract

**Background and objective: **Integrative Medicine and Health (IMH) is a theory-based paradigm shift for health, disease and health care, which can probably only be achieved by supplementing medical roles and competences.

**Definition of IMH:** The definitions of the *Academic Consortium for Integrative Medicine* *and Health 2015* and the so-called *Berlin Agreement: Self-Responsibility and Social Action in Practicing and Fostering Integrative Medicine and Health Globally* are used. The basic features of evidence-based Integrative Medicine and Health (EB-IMH) are based on the recommendations on EBM by David L. Sackett.

**Global State of Undergraduate and Postgraduate Medical Education (UG-PGME) for IMH: **The USA and Canada are most advanced in the development of IMH regarding practice, teaching and research worldwide. Despite socio-cultural peculiarities, they can provide guidance for Europe and especially for Germany. Of interest here are competences for UG-PGME in IMH in primary care and in some specialist disciplines (e.g. internal medicine, gynecology, pediatrics, geriatrics, oncology, palliative care). For these specialties, the need for an interprofessional UG-PGME for IMH was shown in the early stages of development.

**UG-PGME for IMH in Germany: **In the course of the development of the new Medical Licensure Act in Germany (ÄApprO), based on a revision of the National Competence-based Catalogue of Learning Objectives for Medicine (NKLM 2.0) and new regulations for Postgraduate Medical Education in Germany, suggestions for an extension of UG-PGME are particularly topical. To some extent there are already approaches to IMH. Old and new regulations are set out and are partly compared. As a result, some essential elements of IMH are mapped in the new ÄApprO. The new regulations for Postgraduate Medical Education do not mention IMH.

**Conclusion: **The development of medical competences for IMH in the continuum of the UG-PGME could be supported by the coordinated introduction of appropriate entrustable professional activities (EPA) and IMH sub-competences combined with appropriate assessment.

## Zusammenfassung

**Hintergrund und Zielsetzung:** Die Integrative Medizin und Gesundheit (IMG) ist ein theoriegeleiteter Paradigmenwechsel für Gesundheit, Krankheit und Gesundheitswesen, der vermutlich nur durch eine Ergänzung ärztlicher Rollen und Kompetenzen geleistet werden kann.

**Definition von IMG: **Es wird die Definition des „Academic Consortium for Integrative Medicine and Health 2015“ und die sogenannte „Berliner Vereinbarung für Eigenverantwortung und Handlungen der Gesellschaft für eine globale Praxis und Förderung der IMG“ verwendet. Die Grundzüge dieser evidenz-basierten Integrativen Medizin und Gesundheit (EB-IMG) orientieren sich an den Empfehlungen zur EBM von David L. Sackett.

**Globaler Stand der ärztlichen Aus- und Weiterbildung (AuWB) für IMG:** Die USA und Canada sind in der Entwicklung der IMG für Praxis, Lehre und Forschung weltweit am weitesten fortgeschritten. Trotz sozio-kultureller Besonderheiten können sie Orientierungshilfen für Europa und speziell für Deutschland bieten. Von Interesse sind hierbei insbesondere Kompetenzen für eine AuWB in IMG in der Primärversorgung und in einigen Spezialdisziplinen (z. B. Innere Medizin, Gynäkologie, Pädiatrie, Geriatrie, Onkologie, Palliativmedizin). In diesen hat sich früh der Bedarf einer interprofessionellen AuWB gezeigt.

**AuWB für IMG in Deutschland:** Im Zuge der Entwicklung einer neuen ärztlichen Approbationsordnung (ÄAppO), einer dieser zugrunde liegenden Weiterentwicklung des Nationalen Kompetenzbasierten Lernzielkatalogs (NKLM 2.0) und einer neu verabschiedeten (Muster)-Weiterbildungsordnung in Deutschland sind Überlegungen für eine Erweiterung der ärztlichen Aus- und Weiterbildung (AuWB) besonders aktuell. Teilweise gibt es schon Ansätze dafür. Alte und neue Ordnungen werden dargelegt und zum Teil verglichen. Im Ergebnis werden in der neuen ÄAppO wesentliche Elemente der IMG abgebildet. In der Novelle der (Muster-)Weiterbildungsordnung ist die IMG nicht berücksichtigt.

**Schlussfolgerung: **Für die Entwicklung der ärztlichen Kompetenzen für IMG im Kontinuum der AuWB könnte die abgestimmte Einführung entsprechender Anvertraubarer Professioneller Tätigkeiten (APT), IMG-Teilkompetenzen und angemessener Prüfungen geeignet sein.

## Introduction

The roles and competences of physicians (knowledge, skills, values and attitudes) require some important additions when principles of integrative medicine and health (IMH, abbreviations see attachment 1 ) are comprehensively applied in individual patients and in the health care system in comparison with conventional and complementary medicine. After all, IMH is a theory-based paradigm shift for health, disease and the health care system that cannot be achieved with old approaches. Against the background of the emergence of the new Medical Licensure Act in Germany (ÄApprO), based on a revision of the National Competence-based Catalogue of Learning Objectives for Medicine (NKLM 2.0) and new regulations for a competence-based Postgraduate Medical Education (PGME) in Germany, the situation is complex. After a global overview of Undergraduate and Postgraduate Medical Education (UG-PGME) for IMH, proposals for a corresponding extension of UG-PGME in Germany are made in this article, informed particularly through research and development in North America.

## Definition of integrative medicine and health as a basis for undergraduate and postgraduate medical education (UG-PGME)

Following the Berlin Agreement for Self-Responsibility and Social Action in Practicing and Fostering Integrative Medicine and Health Globally [1] the definition of IMH used here is as follows [2]:

Integrative medicine and health reaffirm the importance of the relationship between practitioner and patient, focuses on the whole person, is informed by evidence, and makes use of all appropriate therapeutic and lifestyle approaches, healthcare professionals and disciplines to achieve optimal health and healing.

There are numerous other definitions or descriptions of IMH which, apart from nuances, are essentially not contradictory. The definition of the Academic Consortium (AC) is a framework for action but should ideally be embedded in a larger theoretical framework of IMH. A precursor to the AC definition was elaborated by Boon et al. [3], including concepts for cooperation between health professions and disciplines for integrative health care. Unfortunately, there is currently no globally agreed theory of IMH, and not for health and disease in general.

The World Health Organization placed its own 1948 definition into perspective and replaced it with the “right to the highest attainable standard of health” and explained this in more detail [4]. Attempts are therefore being made to rethink health and disease with their determinants [5], [6] whose theoretical framework is compatible with that of IMH [5]. Such an attempt was also made for Integrative Health (IH) after a survey of stakeholders and experts [7]. The resulting definition is very interesting but has a crucial limitation: it explicitly excludes the criterion “based on scientific evidence”.

The definition of IMH and its application used here refer to evidence-based IM (EB-IM) or evidence-based IMH (EB-IMH). The principles of evidence-based medicine (EBM) [8], [9] as an element of evidence-based health care (EBHC) [10] therefore have a special significance for the practice of IMH and hence for UG-PGME. The principles of EBM and EBHC can be applied directly to IMH and should therefore be quoted here literally:

*Evidence-based medicine (EBM) requires the integration of the best research evidence with our clinical expertise and our patient’s unique values and circumstances *[9]*.*

The last part of EBM as a basis for the inclusion of patient preferences is often overlooked but is of central importance for every type of healthcare and thus also for IMH.

The general aspects of EB-IMH discussed so far come almost exclusively from the Anglo-American cultural area. However, this should not obscure the fact that the concept of IM was already set out in Germany in 1992, including the awareness of a different doctrine that would also be necessary for this new way of thinking and acting [11], [12].

## Integrative medicine and health in undergraduate and postgraduate medical education international

### Standards of the World Federation for Medical Education (WFME)

In 2015, the *World Federation for Medical Education (WFME)* issued standards for undergraduate medical education [13] and standards for postgraduate medical education [14], which are widely used internationally for accreditation purposes. Both standards do not mention IM. Nor is there any theoretical framework for defining health or disease mentioned (see attachment 2 ).

The WHO has repeatedly addressed the importance of traditional medicine and complementary medicine. The most detailed global analysis dates back to 2013 [15]. In the recommended strategy, the term IMH appears only in a cited article. Challenges of UG-PGME in IMH and for traditional medical systems are not addressed.

## Undergraduate and postgraduate medical education for integrative medicine and health in North America

The acquisition of IMH competences in UG-PGME by physicians (and other health professions) has developed most intensively and rapidly in the USA since the founding of the* Andrew Weil Center for Integrative Medicine* at the University of Arizona (USA) in 1994 [16]. This will be presented in more detail in selected examples because it could serve as a guide for the German situation (see attachment 3 ). In particular, the curricula and their learning objectives for UG-PGME can serve as orientation for a wide range of health professions in Germany (see attachment 4 , tables 1-5).

## Undergraduate and postgraduate medical education for integrative medicine and health in Europe (including Russia)

In Europe, including Russia, complementary and alternative medicine (CAM) is widespread, but there are limited approaches to IMH. This is summarized in attachment 5 (excluding Germany).

## Undergraduate and postgraduate medical education for integrative medicine and health in South America, Australia, Asia, Middle East and Africa

This is summarized in attachment 6 , with special interest in looking at China.

## Undergraduate and postgraduate medical education for integrative medicine and health in Germany

### Undergraduate medical education

The current licensing regulations for doctors which dates to 27.06.2002 and entered into force on 01.10.2003 and was last altered 01.03.2020 https://www.gesetze-im-internet.de/_appro_2002/BJNR240500002.html] request in the cross-sectional area 12 “Rehabilitation, Physical Therapy, Naturopathic Medicine” that a proof of performance for naturopathic medicine must be provided. Since 2002, homeopathy has been included as an elective subject without proof of performance. Proof of performance is also requested for social influencing factors and mental-spiritual dimensions of human beings in § 27, 3. “Occupational Medicine, Social Medicine” and § 19. “Psychosomatic medicine and Psychotherapy” as well as in the cross-sectional areas 2: “History, Theory, Ethics”, 3: “Health Economics, Health System, Public Health Care“, and 10: “Prevention, Health Promotion”. IMH is not addressed either conceptually or in terms of content.

On 29.11.2019, the Federal Ministry for Health (BMG) submitted the draft of the new Medical Licensure Act in Germany (ÄApprO), after the so-called Master Plan for Medical Education 2020 was adopted by the relevant political bodies about 3 years ago. Up until the end of January 2020, medical faculties and relevant organizations were invited to submit amendments, based upon which a ministerial legislative proposal will be drawn up and parliamentary procedures and deliberations will begin. The new licensure regulations are expected to enter into force in 2025. 

A summary of the main innovations can be found in the journal Deutsches Ärzteblatt [17]. In the draft, the term “natural healing method” is used twice: in Appendix 2 under “Clinical subjects which are part of the certificates of performance to be provided up to the first, second and third sections of the medical examination” and in Appendix 8 under the elective themes, for which “… proof of performance in accordance with Section 24 (1) must be provided up to the third segment of the medical examination..., if it is offered by the university...”. Also included in this section is homeopathy. The terms “Integrative Medicine/Health”, “Complementary Medicine”, “Alternative Medicine”, “Chinese Medicine”, “Anthroposophical Medicine” and others are not listed there. 

The practice of EBM is particularly important for UG-PGME in IMH. The inaccurate presentation of medical competence for evidence-based medicine in the draft is therefore particularly regrettable. Appendix 15 (super-ordinated, competence-related test material for the first, second and third section of the medical exam), p. 110 states: “Principles and methods of evidence-based medicine and application in patient treatment and in the clinical context”. This is supplemented in § 115 (contents of the fourth section of the medical exam) and in § 116 (content and duration of the exam using patients), p. 55-56 and in Section B. Special part (content and duration of the exam using patients), p. 172. “The evidence-based processing of a clinical issue” is intended, apparently in the form of an “evidence-based case report”. While § 115 mentions some important competences for identifying and assessing a patient’s preferences, values and socio-cultural background, this is not directly related to the practice of EBM [8], [9]. This gives the impression that EBM is (mainly) based on scientific results (“evidence”).

The medical curriculum should be consistently based on the competences of future physicians and comply with the National Competence-based Catalogue of Learning Objectives for Medicine (NKLM) adopted in 2015 by the German Association of Medical Faculties (MFT), initiated by the Association for Medical Education of German speaking countries (GMA) and developed jointly with the MFT [18], [http://www.nklm.de]. However, this provision should apply to an advanced version of the NKLM (NKLM 2.0) which will be created by a working group and will probably be available in 2021.

In the current NKLM, the term Integrative Medicine and Health is not used, but competences are maintained that would indirectly support IMH. The term “naturopathic medicine (naturopathy)” alone is mentioned 59 times across disciplines. Chapter 16 “Therapeutic Principles” under Competence/Learning Objective number 16.9 (see attachment 4 , table 5) lists: “The graduate is able to describe and explain the therapeutic principles of physical medicine, naturopathic medicine, complementary and alternative medical procedures, evaluate them critically and prescribe them adequately where required. He or she can ...”. Several *sub-competences/learning objectives* and examples of *applications* are then identified, which are well suited for the teaching of integrative medicine and health: e.g. *the sub-competence/learning objective 16.9.1.12*: (they are able to) describe the concepts and methods of classical naturopathic medicine and discuss their effectiveness and risks. Examples of application: Classical naturopathic medicine: hydrotherapy, exercise therapy, nutritional therapy, phytotherapy, mind-body therapy, detoxification; complex concepts such as the Kneipp concept, functional movement theory, dietary-oriented cure. For *the sub-competence/learning objective 16.9.1.13*: (they are able to) describe the physiological hypotheses of relevant complementary and alternative medical orientations and discuss their effectiveness and risks. *Examples of applications*: osteopathy, traditional Chinese medicine, neural therapy, anthroposophical medicine, homeopathy.

In addition, learning objectives and numerous application examples for sub-competences of physical medicine, physiotherapy, manual medicine, occupational therapy, thermotherapy and hydrotherapy, electrotherapy and ultrasound therapy, massage therapy, sports therapy, inhalation therapy, phototherapy, balneotherapy and climate therapy are named. Many of these sub-competences/learning objectives are proposed for “Weiterbildungskompetenz (WK)” = Competence to enter PGME at competence level 3b (i.e.: **competence for practice**: to act independently in an appropriate manner as the situation demands and knowing the consequences; see attachment 4 , also the legend of table 5). For the above-mentioned sub-competences/learning objectives 16.9.1.12 and .13, on the other hand, only level of competence 2 is provided for (i.e.: **knowledge of action and justification**: explaining facts and contexts, categorizing them in the clinical-scientific context and evaluating them based on data; see attachment 4 ,also the legend in table 5. This is two levels below 3b and an indication that these examples of application are not seriously considered for medical practice at entry into PGME. 

In the NKLM, reference to EBM is made at several points. However, the NKLM in Appendix B (List of Literature on Learning Objectives) refers to a “Curriculum Evidence-based Medicine in UGME” of the German Network of Evidence-based Medicine (DNEbM e.V.), which refers to the definition of Sackett [8], but in a truncated form. The annotation of EBM by Sackett in this work on p. 71 *“Increased expertise is reflected in many ways, but especially in more effective and efficient diagnosis and in the more thoughtful identification and compassionate use of individual patients’ predicaments, rights, and preferences in making clinical decisions about their care”* is not considered here. Attachment 2 to this curriculum (learning objectives) also does not set this out clearly enough, as is also the case in the glossary of the DNEbM [19]. It mentions only “patient’s views”, and that does not affect the meaning of Sackett’s proposals (see above in “Definition of EBM”), and it ignores a central element of general clinical practice. In the *graduate profile of NKLM 2.0*, among the entrustable professional activities (EPA) for *diagnosis, differential diagnosis* and *therapy* the learning objectives are now defined as *“according to the principles of EbM and clinical decision-making”* (see “PGME” below).

In summary, it is noted that a great step forward would be made in the direction of preparing young doctors for integrative medicine and health compared to the current licensure regulations if the content described above were included in the NKLM 2.0 and used by the faculties in their curriculum. The practice of EBM should refer explicitly to the definition of Sackett [8], [9]. IM and/or IMH are offered in the curriculum at some educational establishments in Germany (see attachment 7 ).

#### Postgraduate medical education 

On 15.11.2018, the Executive Board of the Federal Medical Association (Bundesärztekammer) adopted new Prototype Regulations for Postgraduate Medical Education (MWBO) for physicians, which are competence-based. In certain programs for postgraduate specialization (occupational medicine, public health, forensic medicine, psychosomatics and psychotherapy) elements are included, which can be useful for integrative medicine. The MWBO provides for additional postgraduate training for acupuncture, manual medicine/chirotherapy, homeopathy, naturopathic procedures, physical therapy as well as balneotherapy/medical climatology, which can be considered as elements of IMH. Elements of a practice of medicine aimed at the mental-spiritual dimension of human beings can be found in postgraduate medical education for psychiatric-psychotherapeutic subjects, in psychosomatic medicine and psychotherapy. The term “integrative medicine” does not occur; the principles and practices of the IMH are not considered in PGME. Many therapeutic approaches for IM are not mentioned, or they are taught unconnected, hence complementary, often also alternatively. The terms “evidence-based medicine”, “evidence-based knowledge” and “evidence-based methods” are clearly mentioned only with reference to the evidence from scientific studies. Thus, the practice of evidence-based medicine [9] is not the subject of the novel MWBO! There is a great deal of skepticism about the inclusion of special values in our population, as is shown by the example of homeopathy. Of 14 state chambers of physicians of the federal states of Germany, which have ratified further training regulations based on the novel MWBO, 9 no longer offer homeopathy as an additional postgraduate training (Zusatzweiterbildung): Brandenburg, Hamburg, Schleswig-Holstein, Bremen, Hesse, Mecklenburg-Vorpommern, Lower Saxony, North Rhine-Westphalia, Saxony-Anhalt [as of 16.07.2020]. The postgraduate medical education certificates of homeopathy acquired previously will continue to be valid in the future. 

## Future of undergraduate and postgraduate medical education for integrative medicine and health in Germany

### Undergraduate medical education

The sub-competences/learning objectives in Chapter 16.9 of the NKLM and in the graduate profile offer opportunities for UGME, if these were used for the diagnostic and theoretical characteristics of IMH within the the definition used here. The methods of learning and assessment could be brought together in a longitudinal curriculum; they are not fundamentally different from medical didactic methods for other competences. Since students particularly appreciate the learning situation with real patients [20], these should be used early on. In-patient learning facilities for simulated and real patient care [21] as well as patient conferences involving the relevant health disciplines and professions [22], [23], [24] would be particularly suitable – also for PGME, see below. Such interprofessional and interdisciplinary integrated medical patient conferences have the potential to become a fundamental element in health care analogous to tumor conferences. Attachment 4 sets out tables of competences that could be used to formulate Entrustable Professional Activities (EPT) [25] necessary for the practice of IMH (EPT-IMH). The focus is on the central elements of IMH, such as the practice of EBM, the medical history technique with the aim of integrating patient values and (necessary for every doctor) the interprofessional cooperation. The competences required for interprofessional cooperation (see attachment 4 , table 4) can be made visible on learning wards and at patient conferences for various health professions. This should be required and checked at level 3a (see legend to table 5, attachment 4 ) for admission to PGME programs (graduate profile). Didactical possibilities for acquiring and verifying such EPA-IMH were highlighted in a workshop with international participation [26]. For the NKLM 2.0, the competences required for entering into PGME (graduate profile) are currently being further developed in a working group of the Association of Medical Faculties (MFT) in Germany. EBM as an essential condition for IMH within the meaning of the definition of EBM [9] and EBHC [10] cited above is not (yet) explicitly defined in the draft version of the NKLM 2.0. The attitudes, values and preferences of the patient(s) that are indispensable for participatory decision-making, both for individual patients [27] and for the application of guidelines [28], should be mentioned as learning objectives. For some learning objectives, the preparation of diagnostics, differential diagnostics or therapy planning is defined according to the *“principles of EbM and clinical decision-making”*. The principle of clinical decision-making [29] is very interesting in its relationship with EBM and thus also with the learning objectives of the EPA-IMH. It would be well suited to include such learning objectives in this concept.

#### Postgraduate medical education

Since it is generally uncommon in Germany to use curricula in PGME programs, these would have to be developed including the definition of stages of development (“milestones”). For learning wards and patient conferences see the section on UGME above. The competencies for entrance into PGME (graduate profile) should be trained and assessed at level 3b (see legend to table 5, attachment 4 ) (see also section on **UGME** above).

#### Assessment in undergraduate and postgraduate medical education for integrative medicine and health

For *factual knowledge* – to name and describe descriptive knowledge (facts) – and *knowledge of actions and their justification* – explaining facts and contexts – oral and written assessment must be prepared by the German National Institute for State Examinations in Medicine, Pharmacy and Psychotherapy (IMPP). For the **practice** of IMH, *competence for practice* – a. to carry out and demonstrate under guidance and b. to act independently in an appropriate manner as the situation demands and knowing the consequences – is necessary and accordingly, assessment by observation such as by OSCE, DOPS or mini-CEX etc. must be carried out. The latter would be irreplaceable particularly for the examination of IMH competences during PGME. Since the competences of IMH are particularly relevant for primary care (general medicine, pediatrics, GPs for internal medicine; see attachment 4 , tables 3 and 4), priorities for these specialties should be given in PGME. It is to be hoped that the next amendment of the new regulations for PGME in Germany (MWBO) in general and especially for competencies of EB-IMH will adapt its quality standards to international development [14].

## Competing interests

The author declares that he has no competing interests.

## Supplementary Material

Abbreviations

Standards of the World Federation for Medical Education (WFME)

Undergraduate and postgraduate medical education for integrative medicine and health in North America

Tables 1-5

Undergraduate and postgraduate medical education for integrated medicine and health in Europe (including Russia)

Undergraduate and postgraduate medical education for integrative medicine and health in South America, Australia, Asia, Middle East and Africa

Centers of undergraduate and postgraduate medical education in Germany where integrative medicine and health is taught – concept and terminology
