# Supplementary material for: Integrative medicine and health in undergraduate and postgraduate medical education
Source: GMS J Med Educ. 2021 Feb 15;38(2):Doc46. doi: 10.3205/zma001442 (PMC7958908; doi:10.3205/zma001442)
Supplement: Abbreviations [file JME-38-2-46-s-001.pdf]

Attachment 1: Abbreviations

AC=Academic Consortium for Integrative Medicine and Health

ACGME=Accreditation Council for Graduate Medical Education

ÄApprO=Medical Licensure Act

BMG=Bundesministerium für Gesundheit (Federal Ministry for Health)

CAM=Complementary and Alternative Medicine

DOPS=Direct Observation of Procedural Skills

EPA-IMG=Entrustable Professional Activity (EPA) for Integrative Medicine and Health (IMH)

EBM=Evidence-based Medicine

EBHC=Evidence-based Health Care

EBIM=Evidence-based Integrative Medicine

EBIMH=Evidence-based Integrative Medicine and Health

EPA=Entrustable Professional Activity

EU=European Union

GMA=Association for Medical Education in German speaking countries

IH= Integrative Health

IM=Integrative Medicine

IMH=Integrative Medicine and Health

MFT=Association of Medical Faculties in Germany

MWBO=(Muster-)Weiterbildungsordnung=Prototype of regulations for postgraduate medical education in Germany, set out by the Executive Board of the Federal Medical Association (Bundesärztekammer) to be awarded state recognition by each federal state individually.

mini-CEX=Mini-Clinical Evaluation Exercise

NKLM 2.0=Revised National Competence-based Learning Objectives for Medicine (in Germany)

OSCE=Objective Structured Clinical Examination

PGME=Postgraduate Medical Education

SSP="Situations as Starting Points" (The Joint Commission of the Swiss Medical Schools - The Profiles Working Group 2017)

UG-PGME=Undergraduate and Postgraduate Medical Education

WFME=World Federation for Medical Education

Attachment 1 to: Hahn EG. *Integrative Medicine and Health in Undergraduate and Postgraduate Medical Education.* GMS J Med Educ. 2021;38(2):Doc46. DOI: 10.3205/001442

WPMEC=Postgraduate Medical Education Competence (new in the NKLM 2.0: competency profile at entrance into residency)
