# Supplementary material for: Integrative medicine and health in undergraduate and postgraduate medical education
Source: GMS J Med Educ. 2021 Feb 15;38(2):Doc46. doi: 10.3205/zma001442 (PMC7958908; doi:10.3205/zma001442)
Supplement: Standards of the World Federation for Medical Education (WFME) [file JME-38-2-46-s-002.pdf]

Attachment 2: Standards of the *World Federation for Medical Education (WFME)*

In the global standards for basic medical education [13] in sub-area 2.6. “Program structure, composition and duration” quality development standard Q 2.6.4 recommends: The faculty *should* “describe in the curriculum the interface with complementary medicine”. In the standards for postgraduate medical education [14] in sub-area 2.3. “Program content” it is recommended in basic standard 2.3.10: The institution *must* include in the programme clinical work and relevant theory or experience of “the interface with complementary medicine.” As quality development standard Q 2.3.2 it is recommended that the programme provider(s) should “adjust the content to changing contexts and needs of the health care delivery system.”

The WFME standards require a significant extension for IMH and a corresponding statement for a future health care system with contextual modifications. Under this condition, it would be a good and globally applicable basis for the quality of UG-PGME programs for IMH.
