# Supplementary material for: Integrative medicine and health in undergraduate and postgraduate medical education
Source: GMS J Med Educ. 2021 Feb 15;38(2):Doc46. doi: 10.3205/zma001442 (PMC7958908; doi:10.3205/zma001442)
Supplement: Undergraduate and postgraduate medical education for integrative medicine and health in North America [file JME-38-2-46-s-003.pdf]

Attachment 3: Undergraduate and Postgraduate Medical Education for Integrative Medicine and Health in North America

Due to the extensive publications regarding the development and research in the field of IMH in the north American region, comprehensive material for the theory and practice of IMH and the corresponding UG-PGME is available. This development has been supported and driven in the USA by the National Institutes of Health (NIH), the National Center for Complementary and Integrative Health (NCCIH, as a department of the NIH), various scientific societies and associations [30], effective patrons and sponsors mainly from the private sector and, not least, by increasing dissatisfaction with preventive and therapeutic health care in the US population. Reflecting the mood in and analysis of health care, the report of a summit of the Institute of Medicine of the US National Academies, funded by the Bravewell Collaboration, sets out in Chapter 5, p. 111-132 "Workforce and Education" the future medical competences deemed necessary for IMH [31]. This report plays a central role in the further development, particularly because it describes the core competences previously lacking in UG-PGME, however in general descriptions and with reference to pilot programmes launched at that time (see below). It is striking that it was recommended to include IM mainly in PGME programmes (residencies and fellowships) as the curricula of medical training were very densely packed [31],Victoria Maizes, p. 122).

Undergraduate Medical Education in North America

However, it has subsequently been shown that the core competences for IMH [32] proposed by Kligler et al. and compiled in **Attachment 4, Table 1** can be successfully integrated into undergraduate curricular programs [33], [34]. Acceptance [35], [36] and the effect on subsequent medical activity require further research. Moreover, the most common survey tool used for attitudes to IMH appears to be highly dependent on the cultural context [37]. Meanwhile, the prerequisites for the development of appropriate instruments and curricula with the definition of differentiated competences are so explicit, at least for the Western culture, that learning objectives and outcome-oriented examinations can be formulated for different medical areas (see PGME in North America below).

The proposed skills are also suitable for the development of appropriate entrustable professional activities (EPAs) [25] for IMH as a graduate profile. These would have to be embedded in the corresponding, already formulated graduate core competences for access to the PGME programmes of the accreditation agency Accreditation Council for Graduate

Medical Education (ACGME). These general (without IMH) core EPAs as a prerequisite for entry into PGME (residency and fellowship) [38] have triggered intense discussions in the USA [39], [40], but are ultimately considered essential for improving patient care [41], [42]. The supplementation to these EPAs by IMH (EPA-IMH) is not yet developed in North America. Core competences for integrative primary care provide impetus here, in particular those developed for general practice ("Family Medicine"), see **Attachment 4, Table 3** [43], [44], [45].

Postgraduate Medical Education in North America

Programmes for IMH in the USA have focused mainly on PGME curricula. Particularly, PGME programmes (residencies and fellowships) for general practitioners (family medicine) were examined in detail. The results and effects are currently available in several publications. A working group of the Academic Consortium for Integrative Medicine and Health had initially described core competences for special PGME (fellowship in IM) [43], which are compiled in **Attachment 4, Table 2**. These competences were then further developed and used for PGME in general practice (family medicine residency) and validated [44], [45], [46], [47]. In 2013, the Board of American Physician Specialties (ABPS) established the American Board of Integrative Medicine (ABOIM) to enhance specialized PGME for IMH with a recognized exam. The competences for IMH are based on the guidelines of the Accreditation Council for Graduate Medical Education (ACGME). The corresponding specialist examination of the ABOIM (like all PGME examinations in North America) relies on previous structured PGME programmes accredited and documented by the ACGME with core competencies, milestones and EPAs. Since individual performances are assessed in the structured PGME programmes and results documented, the PGME examinations of the "boards" can be pure knowledge-based examinations. These are carried out (predominantly) in writing and/or orally.

In the USA [48] and Canada [49] a large number of PGME programmes for medical IMH are currently being offered, which are essentially based on the core competences proposed by an AC working group [43]. All these programs have not yet developed core EPAs as a graduate profile for PGME (see UGME in North America).

In contrast, competencies have been developed for entry into PGME directly following UGME (residencies, fellowships) in various disciplines, in particular for primary care (family medicine) [44]. Assessment methods for this were tested [50] and the application

multicentrically piloted and tested [47], [51]. These competencies were based on the accreditation requirements of ACGME before the EPA era.

Interprofessional Undergraduate and Postgraduate Medical Education in North America

Particular attention must be paid to the ability to *organize interprofessional cooperation* for the care of an individual patient, competences for which have been proposed, in particular for UGME [32] and in detail for PGME [43], [44], [52], see **Attachment 4, Tables 3 and 4**. Physicians who want to practice IMH, investigators who want to work about IMH and teachers in programs for IMH should be able to know and appraise all relevant disciplines and treatment systems after appropriate UG-PGME and participation in faculty development programs, but they will always have to involve other health professions when it comes to diagnostics and therapy, as is generally necessary in primary care. This is particularly true for integrative *primary* health care. All health professions that can contribute to IMH therefore need interprofessional meta-competences that go beyond their own discipline. These were proposed by an expert group of the National Center for Integrative Primary Healthcare (NCIPH) and published by Kligler et al. 2015. They are compiled in **Attachment 4**, **Table 4** [53]. The NCIPH has also published drafts of competences and sub-competences for the following disciplines: acupuncture and oriental medicine [54], behavioral medicine [55], chiropractic [56], family medicine [45], internal medicine [57], naturopathy [58], nursing [59], pediatrics [60], pharmacy [61], physician assistants [62], preventive medicine [63] and public health [64].

The Academy of Integrative Health and Medicine [65] and the Andrew Weil Center for Integrative Medicine [16] were among those who have notably committed themselves to acquiring these competences, and they also offer multi-professional PGME as a fellowship.
