# Supplementary material for: Integrative medicine and health in undergraduate and postgraduate medical education
Source: GMS J Med Educ. 2021 Feb 15;38(2):Doc46. doi: 10.3205/zma001442 (PMC7958908; doi:10.3205/zma001442)
Supplement: Tables 1-5 [file JME-38-2-46-s-004.pdf]

Attachment 4: Tables 1-5

Table 1: Core competencies for a curriculum for integrative medicine and health in undergraduate medical education: values, knowledge, attitudes and skills ([32], p. 523-525).

Values
A graduating physician shall demonstrate an understanding of the following:

1. A physician is defined by a philosophy and perspective on health and illness as well as by a set of skills and techniques. This broad perspective will improve outcomes for patients, deepen fulfilment in collegial relationships, and enable the physician to find continuing meaning in his or her work.
2. A physician has a broad definition of professionalism, which allows the health care team to become a healing community that supports and develops wholeness in all relationships, those between colleagues as well as those between physician and patient.
3. A physician recognizes the relevance of feelings, beliefs, life experiences, meaning, and faith to his/her professional behavior. This broadens the nature of physician–patient interaction and shifts the conventional boundaries of physician–patient relationship.
4. A physician is able to recognize the value of his or her own full human experience and to focus and dedicate it to the benefit of patients. Who the physician is as a person is transmitted through his or her work and presence and has a substantive impact on the outcome of the doctor–patient relationship.
5. A physician believes that an ongoing commitment to personal growth is fundamental to the practice of medicine.
6. A physician is able to create a relationship of harmlessness, safety, nonjudgment, and acceptance that enables patients to access their own strengths and direct their own lives.
7. A physician recognizes the pursuit of meaning as fundamental to the process of healing and has the capacity to find meaning in daily work and daily relationships. This capacity allows him/her to accompany patients as they seek and find meaning in the events of their lives.
8. A physician recognizes the multivariate and sometimes unknown factors that influence health and healing.
9. A physician views health and illness as a part of human development that can evoke the potential for personal and social wholeness through the experience of illness and suffering.

Knowledge
A graduating physician shall be able to:

1. Discuss how personal, cultural, ethnic, and spiritual beliefs shape an individual’s interpretation and experience of his or her disease and its treatment.
2. Identify the major strengths and limitations of biomedical knowledge as applied to health care delivery.
3. Give examples of the different ways of knowing about illness and healing.
4. Discuss the distinction between the terms “healing” and “curing.”
5. Describe the distinction between integrative medicine (IM) and CAM.
6. Describe the evidence for mind–body–spirit relationships in illness and health.
7. Describe the prevalence and patterns of CAM use in the patient’s community.
8. Describe the basic concepts of the most commonly used CAM modalities such as chiropractic, herbal and nutritional medicine, and mind–body therapies, and of one or more of the widely used traditional systems of medicine such as Chinese medicine and Ayurvedic medicine, including:

a. Basic definitions/theory/philosophy/history
b. Common clinical applications
c. Potential for adverse effects
d. Current research evidence for efficacy
e. Reputable resources for in-depth information
f. Training/credentialing standards for practitioners
9. Identify potential legal and ethical implications related to the inclusion or the exclusion of CAM modalities in a patient's treatment plan.
10. Identify reputable information resources for CAM and IM in order to support life-long learning.
11. Explain the current status of government regulation of herbal medicines and dietary supplements.

Attitudes
A graduating physician shall be able to demonstrate:

1. A respect for the influence of the patient's personal, cultural, ethnic, and spiritual beliefs on their experience of health and illness and on the patient's clinical decision-making process
2. An awareness of how the physician's own personal, cultural, ethnic, and spiritual beliefs may affect their choice of recommendations regarding patients' treatment decisions.
3. A respect for the strengths and limitations of applying evidence-based medicine principles to the circumstances of an individual patient.
4. A respect for the potential of a variety of healing approaches to be effective for the treatment of certain conditions.
5. An awareness of the importance of self-care both for physician well-being and as a model to promote selfcare in patients.

Skills
A graduating physician shall be able to:

1. Demonstrate an ability to assist patients in developing their own self-care program as part of encouraging active patient involvement in health promotion and clinical decision making.
2. Demonstrate skills to communicate effectively with patients about all aspects of their health and illness including biological, psychological, social, and spiritual as part of comprehensive history taking.
3. Demonstrate skills to communicate effectively:
a. with patients about their use of CAM in a respectful and culturally appropriate manner; and
b. with patients and all members of the interdisciplinary health care team in a collaborative manner to facilitate quality patient care. (The team may include nurses, chaplains, nutritionists, social workers, practitioners of healing systems other than allopathic medicine such as Chinese medicine or chiropractic, etc.)
4. Design a personal self-care program that includes:
a. Learning to assess one's level of stress
b. Implementing a self-care strategy (may include nutrition awareness, self-regulatory techniques, exercise, journaling, creative arts, spirituality, mind–body skills, etc.)
5. Demonstrate an ability to utilize the principles of evidence-based medicine in analyzing integrative medicine approaches, including:
a. developing focused questions regarding the application of IM principles or practices for an individual patient;

b. utilizing databases, peer-reviewed publications, authoritative textbooks, Web-based resources, experiential knowledge of CAM practitioners, and participatory observation to gather relevant information;
c. evaluating the information for scientific quality and clinical relevance;
d. formulating a plan to implement findings in care of an individual patient; and
e. evaluating the outcome of applying IM principles or practices in patient care.

TEACHING AND ASSESSMENT METHODS

Given the divergent nature of CAM therapies and the varying levels of evidence that support their use, the integration of these topics into conventional medical education poses a unique challenge. Innovative educational approaches are required to achieve an effective understanding of the principles and practice of integrative medicine. These approaches demand that educators in this area of medicine develop methods beyond those needed to teach new scientific facts. Three key components for effective implementation of teaching in integrative medicine that are not typically part of medical school curricula at medical schools are

- experiential approaches to facilitate an understanding of complementary and alternative therapies;
- education of medical students in self-care and reflection; and
- faculty development programs to produce educators who have both knowledge and skills in integrative medicine and recognize the importance of self-care and reflection in medical education and practice.

Table 2: Core competencies for a PGME program for integrative medicine and health [43], Attachment 1: Patient care and medical knowledge. A comprehensive set of core competencies can also be found at the Academic Consortium for Integrative Medicine and Health 2012 [65]: Integrative Medicine Clinical Fellowship Core Competencies), available at http://links.lww.com/ACADMED/A181.

Patient Care

The integrative medicine fellow should demonstrate compassionate, appropriate, and effective patient care based on the existing evidence base in integrative medicine for disease prevention, treatment of illness, and health promotion.

1.1. Perform an in-depth integrative medicine assessment.
1.1.1. Demonstrate advanced skill in collecting essential components of an integrative medicine assessment, including but not limited to:
• Identifying patients' health concerns, goals, and expectations.
• A thorough conventional medical history and physical exam.
• Current and past complementary and alternative medicine (CAM) therapy use, including patient experience and response.
• Current and past dietary supplement intake.
• Nutrition, physical activity, sleep pattern.
• Stressors and stress management skills.
• Personal relationships, social network, support systems.
• Religious and spiritual history.
1.1.2. Develop an appropriate differential diagnosis and perform a diagnostic evaluation based on available guidelines and evidence for conventional and integrative testing.
1.2. Demonstrate advanced skills in developing integrative medicine treatment plans based on patient values and preferences, up-to-date scientific evidence, and clinical judgment. Treatment plans should:
1.2.1. Integrate conventional medicine, evidence-based complementary therapies and lifestyle modification, as appropriate.
1.2.2. Address patient concerns in one or more domains (e.g., physical, psychological, social, spiritual).
1.3. Counsel patients on the risks, benefits, and alternatives to an integrative medicine treatment plan, including a discussion of existing evidence to facilitate informed decision making on integrative approaches to care.
1.4. Demonstrate advanced skills in assessment and treatment of patients with complex conditions, including symptoms or diseases with unexplained or poorly understood etiologies.
1.5. Perform competently all medical procedures or complementary medicine therapies appropriate for the fellow's area of practice.*
1.6. Provide health care services aimed at preventing illness and promoting health and wellness.
1.6.1. Collaborate with patients to plan and perform evidence-based health screenings for disease prevention using relevant conventional and integrative approaches.†
1.6.2. Counsel patients on the evidence for integrative therapies for optimizing health and wellness.
1.6.3. Educate patients on the role of lifestyle factors for optimizing health and wellness. Give adequate consideration to sleep, diet, exercise, stress, habits, relationships, community, and spirituality as potential factors influencing health.

1.6.4. Demonstrate expertise in facilitating behavioral changes in patients using evidence-based strategies.‡

2. Medical Knowledge

The integrative medicine fellow should demonstrate advanced knowledge of established and evolving biomedical, clinical, epidemiological, and social-behavioral sciences relevant to integrative medicine.

2.1. Describe the scope and practice of integrative medicine including:
• Domains of complementary and alternative medicine (CAM) as defined by the National Center for Complementary and Alternative Medicine (NCCAM) of the National Institutes of Health.
• Evolving role of CAM in co-management of patients.
• History of integrative medicine.
• Settings in which integrative medicine care is provided.
• Demographic and epidemiologic patterns of integrative medicine use.
• Status of commercial and government insurance coverage for integrative services.
• Legal and ethical issues relevant to the practice of integrative medicine.
• Barriers to access of integrative medicine services.

2.2. Demonstrate expert knowledge of the established and evolving evidence base for how lifestyle factors influence health and illness, including:
2.2.1. The impact of emotional and physical stress on biologic markers and disease processes.
2.2.2. Advanced nutrition theory and practice, such as the role of intensive diet change in the treatment of chronic disease, the impact of medications on micronutrient levels, the role of specific diets for varied diseases, and the safety and evidence for "fad" diets.
2.2.3. Science of physical activity recommendations and required components of individualized exercise prescriptions.
2.2.4. Knowledge of sleep disorders and impairment from sleep deprivation, including their impact on comorbidities and wellbeing.
2.2.5. Role of social connections and spiritual beliefs to health and illness.
2.3. Describe the established and evolving evidence base for common CAM and traditional medical systems, including current information on effectiveness, safety, indications, contraindications, mechanisms, and interactions.
2.4. Describe the established and evolving evidence base for dietary supplements in the management of common medical conditions.
2.4.1. List common uses, potential adverse effects, drug–supplement interactions, clinical pharmacology, and dosing for frequently encountered dietary supplements.
2.4.2. Demonstrate advanced knowledge of the evidence for efficacy and safety of commonly used dietary supplements.
2.4.3. Identify credible evidence-based resources for information on dietary supplements.
2.4.4. Explain historic and current regulations governing dietary supplements in the United States.
2.4.5. Understand the influence of dietary supplement regulations on dietary supplement clinical efficacy, safety, and quality.
2.5. Describe the history, philosophy, and theory for common CAM therapies and traditional medical systems.
2.6. Demonstrate advanced knowledge of the evidence-based integrative medicine model as applied to a range of common clinical conditions.

2.7. Demonstrate advanced knowledge of principles central to integrative medicine practice, including but not limited to:

- Evidence-based medicine.
- Medical pluralism.
- Preventive medicine.
- Patient-centered care.
- Therapeutic alliance.
- Biopsychosocial model and holism.
- Placebo effect.
- Cultural competence.
- Physician self-care.
- Behavioral change.
- Biochemical individuality.
- Patient self-care to enhance resiliency.

* Includes medical interventions or complementary practices, such as acupuncture, relaxation training, and manipulation. Approaches used may be specific to fellow’s subspecialty.
† Examples include personalized approaches to preventive care based on personal and family history, such as preventive cardiovascular testing.
‡ Examples of behavior change theories and techniques include motivational interviewing and the stages of change model.

Table 3: Core competences for integrative health care in general medicine [44], [45]. In the table shown here [45], 24 sub-competences are divided into 10 meta-competences developed for interprofessional integrative health care [53], see **Attachment 4, table 4**. The work of Locke et al. 2013 focuses on general medicine and assigns 19 learning objectives as partial competences to the 6 domains of the Accreditation Council for Graduate Medical Education (ACGME) in order to be compatible with the accreditation system: 1. Patient care; 2. Medical knowledge; 3. Skills of interpersonal relationships and communication; 4. Practice-based learning and improvement; 5. Professionalism; 6. Systems-based practice. In essence, the two proposals are in line.

Family Medicine Competencies DRAFT [78]

1. Practice patient-centered and relationship-based care.
    - Recognize the value of relationship-centered care as a tool to facilitate healing.
    - Demonstrate respect and understanding for patients' interpretations of health, disease, and illness that are based upon their cultural beliefs and practices.
    - Demonstrate the ability to reflect on elements of patient encounters, including personal bias and belief, to facilitate understanding of relationship-centered care.
2. Obtain a comprehensive health history which includes mind-body-spirit, nutrition, and the use of conventional, complementary and integrative therapies and disciplines.
    - Demonstrate patient-centered history taking, using a biopsychosocial approach that includes an accurate nutritional history, spiritual history, and inquiry of conventional and complementary treatments.
3. Collaborate with individuals and families to develop a personalized plan of care to promote health and well-being which incorporates integrative approaches including lifestyle counselling and the use of mind-body strategies.
    - Collaborate with patients in developing and carrying out a health screening and management plan for disease prevention and treatment using conventional and complementary therapies when indicated.
4. Demonstrate skills in utilizing the evidence as it pertains to integrative healthcare.
    - Understand the evidence base for the relationships between health and disease and the following factors: emotion, stress, nutrition, physical activity, social support, spirituality, sleep, and environment.
    - Evaluate the strengths and limitations of evidence-based medicine (EBM) as it applies to conventional and complementary approaches and its translation into patient care.
    - Use EBM resources, including those related to CAM, at the point of care.
    - Identify reputable print and/or online resources on conventional and complementary approaches.

5. Demonstrate knowledge about the major conventional, complementary and integrative health professions.
    - Understand national and state standards related to training, licensing, accreditation, and reimbursement of community CAM practitioners.
    - Demonstrate understanding of common complementary medicine therapies, including their history, theory, proposed mechanisms, safety/efficacy profile, contraindications, prevalence, and patterns of use.
6. Facilitate behavior change in individuals, families and communities.
    - Facilitate health behavior changes in patients, using techniques such as motivational interviewing or appreciative inquiry.
7. Work effectively as a member of an interprofessional team.
    - Demonstrate respect for peers, staff, consultants, and CAM practitioners who share in the care of patients.
    - Collaborate with community CAM practitioners and other healthcare specialists in the care of patients, while understanding legal implications and appropriate documentation issues.
8. Engage in personal behaviors and self-care practices that promote optimal health and wellbeing.
    - Understand importance of self-care practices to improve personal health, maintain work-life equilibrium, and serve as a role model for patients, staff, and colleagues.
9. Incorporate integrative healthcare into community settings and into the healthcare system at large.
    - Understand different reimbursement systems and their impact on patient access.
    - Identify strategies for facilitating access to Integrative Medicine services for their patients, including low-income populations.
    - Understand the principles of designing a healthcare setting that reflects a healing environment.
10. Incorporate ethical standards of practice into all interactions with individuals, organizations and communities.
    - Demonstrate an understanding of ethical principles regarding decisions and treatment that have potential ethical implications including patient autonomy.
    - Act as an effective patient advocate with other members of the health care team
    - Advocate for equal access to all types of therapeutic options regardless of socioeconomic status.
    - Recognize the importance of informed consent and patient awareness of available conventional treatment options when patients are making unconventional choices regarding their health care.
    - Demonstrate personal ethical standards: understand and avoid potential ethical conflicts with the pharmaceutical industries, third-party payers, and other health industry providers, as well as in personal conduct with patients, staff, and colleagues.
    - Demonstrate awareness of limitations in expertise, operate within the jurisdictional scope of practice, and refer care when appropriate.

Table 4: Meta-competences for interprofessional integrative primary care [52], [53]. The comprehensive development and description of these core competencies can be found in Kligler et al. 2015 [52].

1. Practice patient-centered and relationship-based care.
2. Obtain a comprehensive health history which includes mind-body-spirit, nutrition, and the use of conventional, complementary and integrative therapies and disciplines.
3. Collaborate with individuals and families to develop a personalized plan of care to promote health and well-being which incorporates integrative approaches including lifestyle counselling and the use of mind-body strategies.
4. Demonstrate skills in utilizing the evidence as it pertains to integrative healthcare.
5. Demonstrate knowledge about the major conventional, complementary and integrative health professions.
6. Facilitate behavior change in individuals, families and communities.
7. Work effectively as a member of an interprofessional team.
8. Engage in personal behaviors and self-care practices that promote optimal health and wellbeing.
9. Incorporate integrative health care into community settings and into the health care system at large.
10. Incorporate ethical standards of practice into all interactions with individuals, organizations and communities.

Table 5. Competences and learning objectives related to physical medicine, naturopathic procedures and complementary and alternative medicine procedures from **Chapter 16 Therapeutic Principles**, **Subchapter 16.9** of the NKLM [http://www.nklm.de].

ID=identification number; **B**=Basics; **BC**= (medical) basic competence; **PY**=PY competence (for elective clerkship, 6th year of UGME curriculum); **GP**=PGME entrance competence; **SC**=Science competence.

According to the theoretical understanding used here, competence cannot be equated with practical skills or abilities alone. Achieving competence for action (competence levels 3a or 3b) requires the acquisition of factual knowledge (competence level 1) or knowledge about action and justification (competence level 2).
The taxonomy used here to describe the levels of competence has been developed in an international context.

The numbers **2, 3a and 3b** refer to the levels of competence to be achieved, which build on each other:
2. *Knowledge of practice and justification***:** explain facts and contexts, integrate them into the clinical-scientific context and evaluate them based on data.
3. *Competence for practice:*
3a. perform and demonstrate personally under guidance.
3b. carry out independently and appropriately in the knowledge of the consequences.

Level **1.** (*factual knowledge:* name and describe descriptive knowledge (facts).) does not appear here, because the factual knowledge for chapter 16 or sub-chapter 16.9 is defined in other chapters.

Explanation on the meaning of the identification numbers in the NKLM:
Within the sub-chapters, a three-stage, hierarchical structure was used:

- Level 1: Competences
- Level 2: Sub-competences
- Level 3: Learning objectives with indication of competence levels

Levels 1 and 2 are recommended. Level 3 is intended to be used by the medical faculties to be tested and critically evaluated.

The identification number (ID) of the (sub-) competences and learning objectives provides information about the sub-chapter and the respective level of detail. The first digit indicates the respective sub-chapter. The number of the following digits refers to the level of classification.

For example, ID 16.9 denotes a competence in sub-chapter 16.9; 16.9.1.1 denotes a learning objective (level 3) in sub-chapter 16.9; level 2, which denotes sub-competences, is not diversified in sub-chapter 16.9. (therefore, the third figure is always 1).

ID	Competence/ Learning objective resp.	B	BC	EC	PGME	SC	Example of use	Proof of performance according to ÄApprO [sorted for print version]
16.9	The graduate is able to describe and explain the therapeutic principles of physical medicine, naturopathic procedures, complementary and alternative medical procedures, critically evaluate them and, if necessary, prescribe them appropriately. They are able to...							Internal medicine; medicine of aging and the elderly; physics for physicians and physiology; rehabilitation, physical medicine, naturopathic procedures (naturopathic medicine)
16.9.1.1	explain the basic principles of action and the qualities of action of physical medicine			2			Stimulus response principle, influence on organ systems by movement therapy, inhibition and facilitation as well as habituation, relief and protection, sensorimotor adaptation, functional adaptation, trophic-plastic adaptation, behavior modification; immediate and serial effect; kinetic, mechanical, thermal, electrical, actinic, physiochemical quality of action	Gynecology, obstetrics; medicine of aging and the elderly; Physics for physicians and physiology; rehabilitation physical medicine, natural healing methods
16.9.1.2	explain the principles of therapy planning of physical medicine under preventive, curative and rehabilitative objectives, discuss their effectiveness and risks and apply them in an indication-related manner.		2	3a	3b		Therapy planning within the framework of a treatment plan; patient's resilience; differentiated selection, dosage and combination of remedies; method- and indication-related therapy control	Gynecology, obstetrics; medicine of aging and the elderly; Physics for physicians and physiology; rehabilitation physical medicine, natural healing methods
16.9.1.3	describe the principles of physiotherapy, give examples of their methods, therapies and indications and apply criteria for indication		2	3a	3b		Passive measures, active exercise therapy, physiotherapy on a neurophysiological basis, movement therapy with physiotherapeutic modalities, physiotherapy with functional bandages, respiratory therapy, relaxation therapy	Gynecology, obstetrics; medicine of aging and the elderly; physics for physicians and physiology; rehabilitation physical medicine, natural healing methods
16.9.1.4	describe the principles of occupational therapy, give examples of their methods, therapies and indications and apply criteria for indication		2	3a	3b		Functional training of disturbed sensorimotor, neuropsychological or psychosocial functions, self-help training, auxiliary health supply, joint protection, distractive exercise treatment, adaptation of the home and professional environment, occupational therapy	Medicine of aging and the elderly; rehabilitation, physical medicine, natural healing methods (naturopathic medicine)
16.9.1.5	describe the principles of medico-mechanics and apply examples of their methods, therapeutic agents and indication criteria.		2	3a	3b		Functional treatment with mechanical therapy equipment and aids: auxiliary devices, mechanical extension treatment, passive movement splints, walking aids, orthoses	Medicine of aging and the elderly; rehabilitation, physical medicine, natural healing methods (naturopathic medicine)
16.9.1.6	describe the concepts and methods of manual therapy, discuss their effectiveness and risks, and apply indication criteria.		2	3a	3b		Soft tissue techniques, mobilizing joint treatment, neuromuscular therapy, manipulation; concepts such as Maitland, Cyriax, other osteopathic procedures	Medicine of aging and the elderly; rehabilitation, physical medicine, natural healing methods (naturopathic medicine)
16.9.1.7	describe the principles of electrotherapy and therapy with ultrasound and apply examples of their methods, therapeutic agents and indication criteria.		2	3a	3b		Direct-, low-frequency and middle-frequency current therapy, (hydrogalvanic baths, iontophoresis, TENS, electromyostimulation); high-frequency therapy;	Medicine of aging and the elderly; rehabilitation, physical medicine, natural healing methods (naturopathic medicine)

							therapies with ultrasound, phonophoresis	
16.9.1.8	describe the concepts and methods of hydrotherapy and thermotherapy, discuss their effectiveness and risks, and apply indication criteria.		2	3a	3b		Hydrotherapy: washing, wrapping,<br>affusions, baths, rubdown, vapors;<br>cryotherapy: ice, peloid, cold air, spray, cold chamber;<br>thermal therapy: wet packs, hot roll, hot air	Medicine of aging and the elderly;<br>rehabilitation, physical medicine,<br>natural healing methods (naturopathic medicine)
16.9.1.9	describe the principles of massage therapy including manual lymphatic drainage and apply examples of their methods, therapies and indication criteria.		2	3a	3b		Classic massage, reflexology, mechanic massage, lymphatic drainage; concept of<br>Complex Physical Decongestion therapy (CPD)	Medicine of aging and the elderly;<br>rehabilitation, physical medicine,<br>natural healing methods (naturopathic medicine)
16.9.1.10	describe the principles of sports therapy and apply examples of their methods, therapies and indication criteria.		2	3a	3b		Medical training therapy, equipment training; endurance training, strength training, speed training,<br>mobility training,<br>coordination training	Medicine of aging and the elderly;<br>rehabilitation, physics for physicians and physiology;<br>natural healing methods (naturopathic medicine)
16.9.1.11	know the principles of inhalation therapy, phototherapy, balneal and climate therapy and give examples of their methods and indications.		2				Heliotherapy, infrared therapy, light therapy, UV therapy, laser therapy; regional cures; climate therapy, thalassotherapy	Clinical pharmacology/ pharmacotherapy;<br>medicine of aging and the elderly;<br>rehabilitation, physical medicine,<br>natural healing methods (naturopathic medicine)
16.9.1.12	describe the concepts and methods of classical naturopathic medicine and healing methods and discuss their effectiveness<br>and risks.		2				Classical naturopathic medicine and healing methods: hydrotherapy, movement therapy, nutritional therapy, phytotherapy, mind-body therapy, de-toxification procedures; complex concepts such as Kneipp concept, functional movement theory, dietetic orientated cure	Gynecology, obstetrics; medicine of aging and the elderly;<br>rehabilitation, physical medicine,<br>natural healing methods (naturopathic medicine)
16.9.1.13	describe the hypotheses of physiological action of relevant complementary and alternative medicine directions of thought and discuss their effectiveness and risks.		2				Osteopathy, traditional Chinese<br>medicine, neural therapy, anthroposophical medicine, homeopathy	Gynecology, obstetrics; human genetics; medicine of aging and the elderly;<br>rehabilitation, physical medicine, naturopathic procedures (naturopathic medicine)
