# Supplementary material for: Integrative medicine and health in undergraduate and postgraduate medical education
Source: GMS J Med Educ. 2021 Feb 15;38(2):Doc46. doi: 10.3205/zma001442 (PMC7958908; doi:10.3205/zma001442)
Supplement: Undergraduate and postgraduate medical education for integrated medicine and health in Europe (including Russia) [file JME-38-2-46-s-005.pdf]

Attachment 5: Undergraduate and Postgraduate Medical Education for Integrated Medicine and Health in Europe (including Russia)

The use and acceptance of CAM in the European Union (EU) is very widespread, well studied and has been used in particular by the Cambrella project on recommendations for the EU [66], [67], [68], [69]. However, these concerned future health care and research aspects. Concepts and recommendations for UG-PGME in IMH were not addressed; the term IMH was not yet widespread in Europe. In Sweden, there have been considerations for integrative primary care [70], which also includes the need for appropriate PGME for specialists, but compared to North America, no innovative UG-PGME for IMH has developed in Europe's health care systems that correspond to the conceptual framework of the AC definition. The individual therapies and directions of thought of CAM have taken this into their own hands, whereas the medical faculties have (to this day) behaved very skeptically and even negatively. In Russia, traditional and CAM therapies are widely used, also in addition to conventional medicine, but UG-PGME in IMH is not recognizable [71]. The use of CAM by physicians has been and still is consistently regulated differently in the 28 EU countries [72], which further complicates the development of a uniform IMH.

A special situation has arisen for CAM in Switzerland, where after a 70% majority in a referendum in 2009 the parliament decided to introduce a CAM constitutional article into the Federal Constitution (Art. 118a: "The Confederation and the cantons ensure that complementary medicine is taken into account within the scope of their competences."). [https://www.admin.ch/opc/de/classified-compilation/19950275/index.html]. After a 2015 report by the Swiss Drug Administration, in the provision on benefits in compulsory health insurance (KLV of 29 September 1995, as at July 1st, 2020, Chapter 1, 5th section: complementary medical services by physicians), acupuncture, anthroposophical medicine, drug therapy of classical Chinese medicine, classical homeopathy, and phytotherapy were included [https://www.admin.ch/opc/de/classified-compilation/19950275/index.html]. The inclusion of medical complementary medicine in teaching and research was defined by an amendment of the Medical Professions Act 2016 as well as in the 2017 Swiss Catalogue of Learning Objectives for Undergraduate Medical Education (see below). As of 1.1.2017, it became mandatory for universities/schools of higher education to incorporate these different

disciplines of CAM into undergraduate programmes for *all* health professions of the Medical Professions Act. The term IMH was not used. This legislative process as a result of the referendum is clearly presented by Albonico [73]. Patient care, medical teaching and research are increasingly developing in Switzerland under the name IMH. The Institute for Complementary and Integrative Medicine of the Medical Center of the University of Zurich plays a leading role here [74], [75].

The above-mentioned Swiss Catalogue of Learning Objectives for Medical Education, which was published in a 3rd edition in English in 2017 [http://www.profilesmed.ch] does not mention the term IMH. References to complementary medicine and the need to get information about and consider the relevant preferences of the individual patient are included. Also, in two EPA CAM is included (EPA 1.9: *Explore the patient's use of medicine and treatment, including complementary and alternative medicine*, EPA 7.3: *Adopt a shared decision-making approach in establishing the management plan, take into account patient's preferences in making orders, take into account an indication or request for complementary medicine* …). And in the chapter "*Situations as Starting Points* (SSP)" reference is made to CAM: SSP 262: *Benefits and risks of complementary medicine*; SSP 263: *Suspicion of drug intolerance or interaction (including complementary medicine).*
