# Supplementary material for: Integrative medicine and health in undergraduate and postgraduate medical education
Source: GMS J Med Educ. 2021 Feb 15;38(2):Doc46. doi: 10.3205/zma001442 (PMC7958908; doi:10.3205/zma001442)
Supplement: Undergraduate and postgraduate medical education for integrative medicine and health in South America, Australia, Asia, Middle East and Africa [file JME-38-2-46-s-006.pdf]

Attachment 6: Undergraduate and Postgraduate Medical Education for Integrative Medicine and Health in South America, Australia, Asia, Middle East and Africa

In these regions of the world there is a strong use of traditional medicine and KAM [15], [76], usually in addition or with integration into conventional medicine in the respective culture [77]. On the other hand, IMH according to the definition of the AC cannot be established and consequently no UG-PGME for IMH. In countries where tradition was based on an independent complete medical system, such as China and other Asian cultures, a fusion of Western biopsychosocial medicine with traditional medicine and CAM is most likely to be expected. The development in China towards IMH can also be seen in the fact that two journals in China have renamed themselves accordingly: the Journal of Chinese Integrative Medicine is now called Journal of Integrative Medicine (publisher: Elsevier) and the Chinese Journal of Integrative Traditional and Western Medicine is now called Chinese Journal of Integrative Medicine (publisher: Springer). Care, research and UG-PGME usually remain separate in terms of content according to the different therapeutic directions, or a type of traditional medicine in connection with Western medicine is considered. UG-PGME is also published accordingly, but not with a view to a comprehensive IMH.
