# Supplementary material for: Integrative medicine and health in undergraduate and postgraduate medical education
Source: GMS J Med Educ. 2021 Feb 15;38(2):Doc46. doi: 10.3205/zma001442 (PMC7958908; doi:10.3205/zma001442)
Supplement: Centers of undergraduate and postgraduate medical education in Germany where integrative medicine and health is taught – concept and terminology [file JME-38-2-46-s-007.pdf]

Attachment 7: Centers of Undergraduate and Postgraduate Medical Education in Germany where Integrative Medicine and Health is Taught – Concept and Terminology.

Hospital for Integrative Medicine and Naturopathy at the University of *Duisburg-Essen* (https://www.uni-due.de/naturheilkunde/44-0-Curriculum.html).

University Medicine Berlin Charité (with two professorships in naturopathy, https://epidemiologie.charite.de/forschung/projektbereich_komplementaere_und_integrative_medizin/arbeitsgruppe_naturheilkunde_und_integrative_medizin/ and https://epidemiologie.charite.de/forschung/projektbereich_komplementaere_und_integrative_medizin/arbeitsgruppe_klinische_naturheilkunde/ and an (endowed) professorship Integrative Medicine and Anthroposophy, https://epidemiologie.charite.de/metas/person/person/address_detail/matthes/).

Academic Center for Complementary & Integrative Medicine (AZKIM) (a consortium of the Universities of Freiburg, Heidelberg, Tübingen and Ulm, available at http://www.azkim.de/). The declared goal of AZKIM is to establish a well-founded Integrative Medicine (school medicine in harmony with complementary medicine) in Baden-Württemberg, Germany in accordance with the "WHO Traditional Medicine Strategy 2014-2023". The Universities of Heidelberg (Immunology), Freiburg (Naturopathic Medicine, Internal Medicine and Dermatology), Tübingen (General Medicine and Health Systems Research) and Ulm (Pharmacology) cooperate with this goal in patient care, research, UG-PGME in a suitable organisational structure (see also under http://www.azkim.de/ueber-azkim/das-azkim-konsortium/organisationsstruktur)

University of Witten/Herdecke (https://www.uni-wh.de/gesundheit/department-fuer-humanmedizin/lehrstuehle-institute-und-zentren/institut-fuer-integrative-medizin-ifim/)

(Endowed) professorship for Integrative Medicine and Naturopathic Medicine at the *Division for Integrative Medicine and Naturopathy* at Bamberg Hospital, where the teaching is currently being developed and momentary takes place predominantly at the University of Duisburg-Essen.

On closer inspection of the offered curricula it is shown that, if the term IM is used, it is understood as a combination of conventional medicine and CAM. IM is usually conceptually

supplemented by CAM or naturopathy, as if it were to be separated from it. This makes no sense, and IM or IMH understood in this way does not correspond in the final consequence to the IMH set out in the definition used in this article. These terms have been controversial in the past [78]. David Rakel and Andrew Weil also emphasise in their definition of IMH: *"Complementary and alternative medicine (CAM) are not synonymous with integrative medicine"* [79]. This finding is important for IMH in UG-PGME because, until recently, it can be observed that the term "integrative medicine" covers the most diverse CAM disciplines, but not IM itself. This applies both to national and international meetings on "IMH" as well as to research organisations and scientific journals which have recently supplemented or replaced their complementary medical character with the addition of the term "integrative medicine". This is understandable, because previously neglected health professions, disciplines and schools of thought have a legitimate interest in a fair evaluation, both individually and for IMH. IMH, however, requires a new, coherent, precisely integrative thinking and acting from the first patient encounter, in the drafting of research questions and, of course, also in UG-PGME. This is, according to the definition used in this article, the only way to speak of IMH. The spread of this way of thinking as any paradigm shift for a future health care system places high demands on the activities of physicians as well as other health professions.
